# Exposure to Microcystin-LR Promotes Colorectal Cancer Progression by Altering Gut Microbiota and Associated Metabolites in APC^min/+^ Mice

**DOI:** 10.3390/toxins16050212

**Published:** 2024-04-30

**Authors:** Yuechi Song, Xiaochang Wang, Xiaohui Lu, Ting Wang

**Affiliations:** Department of Cell Biology, School of Basic Medical Sciences, Nanjing Medical University, 101 Longmian Avenue, Nanjing 211166, China; songyuechi@njmu.edu.cn (Y.S.); xiaochang@stu.njmu.edu.cn (X.W.); lvxiaohui222118@stu.njmu.edu.cn (X.L.)

**Keywords:** microcystin-LR, Apc^min/+^ mice, colorectal cancer, gut microbiota, bile acid

## Abstract

Microcystins (MCs), toxins generated by cyanobacteria, feature microcystin-LR (MC-LR) as one of the most prevalent and toxic variants in aquatic environments. MC-LR not only causes environmental problems but also presents a substantial risk to human health. This study aimed to investigate the impact of MC-LR on APC^min/+^ mice, considered as an ideal animal model for intestinal tumors. We administered 40 µg/kg MC-LR to mice by gavage for 8 weeks, followed by histopathological examination, microbial diversity and metabolomics analysis. The mice exposed to MC-LR exhibited a significant promotion in colorectal cancer progression and impaired intestinal barrier function in the APC^min/+^ mice compared with the control. Gut microbial dysbiosis was observed in the MC-LR-exposed mice, manifesting a notable alteration in the structure of the gut microbiota. This included the enrichment of *Marvinbryantia*, *Gordonibacter* and *Family_XIII_AD3011_group* and reductions in *Faecalibaculum* and *Lachnoclostridium*. Metabolomics analysis revealed increased bile acid (BA) metabolites in the intestinal contents of the mice exposed to MC-LR, particularly taurocholic acid (TCA), alpha-muricholic acid (α-MCA), 3-dehydrocholic acid (3-DHCA), 7-ketodeoxycholic acid (7-KDCA) and 12-ketodeoxycholic acid (12-KDCA). Moreover, we found that *Marvinbryantia* and *Family_XIII_AD3011_group* showed the strongest positive correlation with taurocholic acid (TCA) in the mice exposed to MC-LR. These findings provide new insights into the roles and mechanisms of MC-LR in susceptible populations, providing a basis for guiding values of MC-LR in drinking water.

## 1. Introduction

Microcystins (MCs), the environmental toxin generated by cyanobacteria [1,2], feature microcystin-LR (MC-LR) as their common and highly poisonous structural variant [3,4]. Acknowledged as a potent hepatotoxin [5,6,7] and a potential oncogenic facilitator [8,9], MC-LR induces hepatic damage in humans [10], livestock and aquatic organisms [11]. The World Health Organization (WHO) stipulates that the permissible level of its presence in drinking water should not exceed 1 μg/L [12]. Nevertheless, burgeoning research suggests that in specific countries like Vietnam [13] and China [14], the concentration of MC-LR in drinking water significantly exceeds the 1 μg/L limit. In 2010, the International Agency for Research on Cancer (IARC) classified MC-LR as a possible human carcinogen (Group 2B) [15]. MC-LR can easily enter cells via specific organic-anion-transporting polypeptides (OATPs) and then bind to certain protein phosphatases with a high affinity, leading to excessive protein phosphorylation, activation of downstream signaling pathways and production of various toxic effects [16,17,18]. Water contaminated with MC might contribute to the heightened incidence of primary human liver tumors in China [19]. While the hepatotoxic effects of MC-LR have been extensively documented, its impact on organs such as the gastrointestinal tract remains less elucidated. Studies indicate that MC-LR exacerbates colitis in mice induced by dextran sulfate sodium (DSS), with negligible effects on the gastrointestinal tract of normal mice [20,21]. This suggests that individuals with pre-existing gastrointestinal conditions are more susceptible to MC-LR. Additionally, research highlights the capacity of MC-LR to promote tumor progression, including breast cancer cell migration [22] and melanoma cell invasion [23]. Given the role of the intestines as the primary site for MC-LR absorption, its potential facilitation of malignant progression in colorectal carcinogenesis is pronounced.

Colorectal cancer (CRC) exhibits a globally elevated incidence and mortality rate [24,25], and several studies have demonstrated that MC-LR acts as an inducer for the occurrence and development of CRC [26,27]. Consistently, our previous research demonstrated the promotion of MC-LR in the migration and invasion of CRC cells [28,29]. Recently, studies showed that the intestinal microbiota may also serve as a significant promotor in the occurrence and progression of CRC [30,31,32]. Research has revealed disparities in the tissue-associated intestinal microbiota between colorectal cancer patients and healthy volunteers [33]. Furthermore, relevant studies have reported that MC-LR also has a certain impact on the homeostasis of intestinal microbes. Studies in animal experiments have indicated substantial impacts of MC-LR on the intestinal microbial community [34,35,36], leading to an elevated presence of pathogenic bacteria and a decrease in beneficial bacteria.

In addition to the carcinogenic effects of gut microbiota dysbiosis, the impact of the intestinal microbiota on CRC is closely linked to the metabolic functions of the microbial community [37,38]. Recent data suggested that microbial metabolites promote the progression of CRC, especially bile acids (BAs) [39,40]. Furthermore, numerous studies have demonstrated the involvement of secondary bile acids in the pathogenesis of CRC, including promoting cell proliferation [41], inhibiting apoptosis of CRC cells [42], promoting P53 degradation [43] and increasing cell aggressiveness [44]. However, research on the impact of MC-LR through the regulation of the intestinal microbiota, influencing microbial metabolism and consequently promoting colorectal cancer progression remains limited.

In this study, our focus centered on elucidating the influence of MC-LR on the gut microbiota and identifying the metabolic byproducts that collaboratively contribute to its effects. Using multi-omics integrated analyses, we aimed to identify microbial species and corresponding metabolites that may potentially facilitate the progression of colorectal cancer. This investigation is intended to serve as a foundational reference for subsequent in-depth exploration into the synergistic promotion of CRC progression by MC-LR and the gut microbiota.

## 2. Results

### 2.1. MC-LR Promoted the Colorectal Tumor Progression and Impaired Intestinal Barrier Function in APC^min/+^ Mice

**Figure 1:**
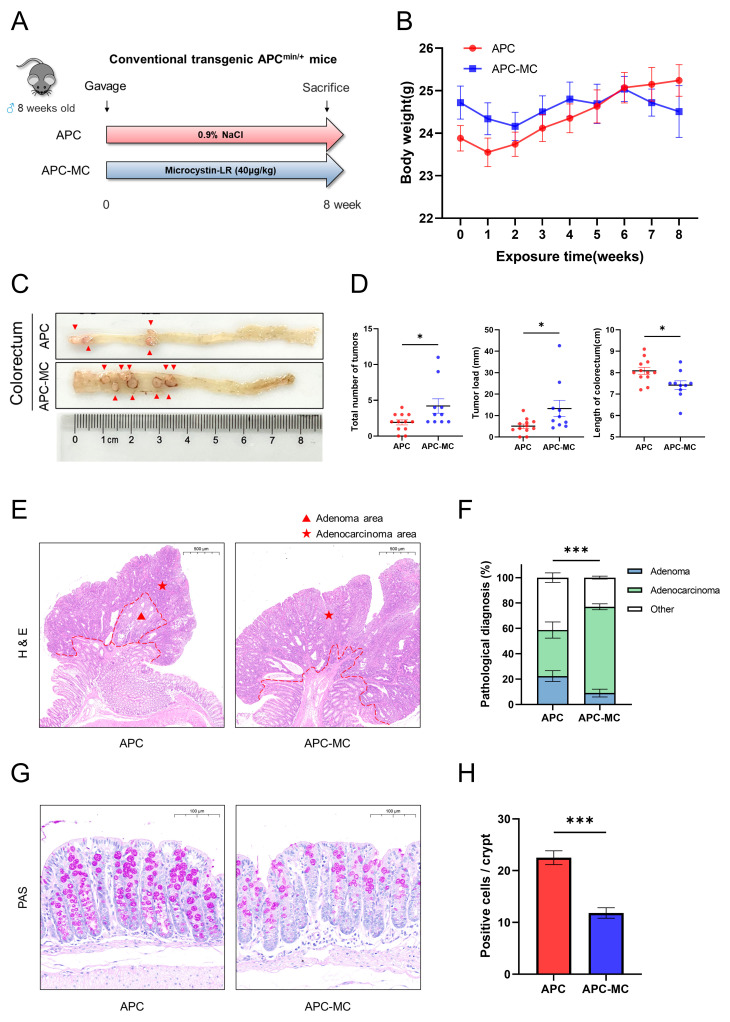
MC-LR promoted the colorectal tumor progression and impaired intestinal barrier function in APC^min/+^ mice. (**A**) The workflow diagram of mice experiment. (**B**) Changes in body weight of mice from week 0 to week 8. (**C**) Representative colon images of the colorectum. (**D**) Tumor number, tumor load and length of colorectum in control and MC-LR-exposed groups. (**E**) Images of H&E-stained areas in the adenoma and adenocarcinoma areas of the control and MC-LR-exposed groups (scale bars: 500 μm). (**F**) The percentage of adenoma area and adenocarcinoma area of mice colorectum. (**G**) Representative images of PAS staining (scale bars: 100 μm). (**H**) The number of colon goblet cells was evaluated by PAS staining. * *p* < 0.05, *** *p* < 0.001.

To assess the influence of MC-LR on the progression of colorectal tumors, transgenic APC^min/+^ mice were subjected to exposure to either a 0.9% NaCl solution or MC-LR (Figure 1A). Throughout the experiment, two mice were observed to die during the MC-LR exposure. In the MC-LR-exposed group, anal prolapse occurred from the 6th week. The mean body weight of the mice in the MC-LR-exposed group exhibited a slight decrease from week 6 to week 8 in comparison to the control. However, no significant difference in body weight was observed between the MC-LR-exposed and control groups (Figure 1B). A few scattered small polyps were observed in the control group, while the MC-LR-exposed group manifested a higher incidence of tumors, notably large adenomas in the colon (Figure 1C). Exposure to MC-LR resulted in a significant increase in both colorectal tumor number and load, coupled with a reduction in colorectal length (Figure 1D). Pathologists validated the presence of colorectal adenomas and adenocarcinomas through histopathological examination (Figure 1E). Exposure to MC-LR resulted in a substantial increase in the area occupied by colorectal adenocarcinoma (Figure 1F).

To conduct a more comprehensive assessment of intestinal barrier damage, staining analysis of goblet cells was performed on colon tissue. The results from the PAS staining indicated a diminished overall count of goblet cells in the MC-LR-exposed group in contrast to the control group (Figure 1G). Statistical outcomes revealed that the mean number of positive cells in each crypt was markedly lower in the MC-LR-exposed group (Figure 1H). These results suggested that MC-LR induced impairment to the intestinal barrier and promoted malignant progression of colorectal cancer tumors in the APC^min/+^ mice.

### 2.2. MC-LR Exposure Did Not Induce Changes in the Alpha Diversity of the Intestinal Microbial Community

**Figure 2:**
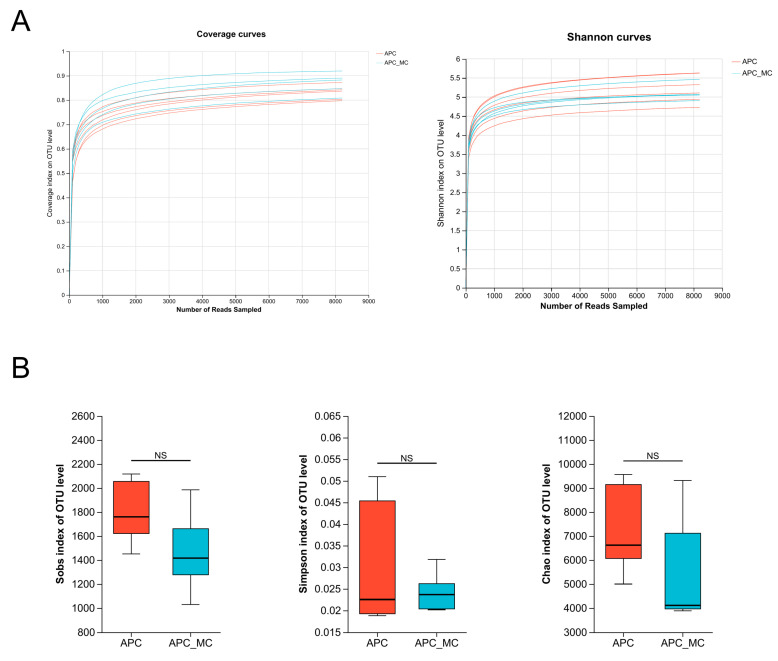
MC-LR exposure did not induce changes in the alpha diversity of the intestinal microbial community. (**A**) Coverage and Shannon curves in the gut microbial community. (**B**) Alpha diversity was compared between control and MC-LR-exposed groups using Sobs, Simpson and Chao index. NS, not significant.

We performed full-length 16S sequencing analysis of fecal samples to determine potential alterations in the gut microbiota by MC-LR exposure. Alpha diversity analysis was primarily employed to evaluate the abundance and diversity of the gut microbiota. The flattening of the rarefaction curve indicated that our sequencing volume adequately covered all the species present in the samples (Figure 1A). We compared the relative abundance of microbial communities between the MC-LR-exposed group and the control group. According to the Chao, Sobs and Simpson index, the abundance and diversity of the intestinal flora in the MC-LR-exposed group showed a decreasing trend, but there was no significant difference between the two groups (Figure 2B). These results indicated that MC-LR exposure did not have an impact on the community diversity of the gut microbiota in the APC^min/+^ mice.

### 2.3. Exposure to MC-LR Altered the Structure of the Gut Microbiota

**Figure 3:**
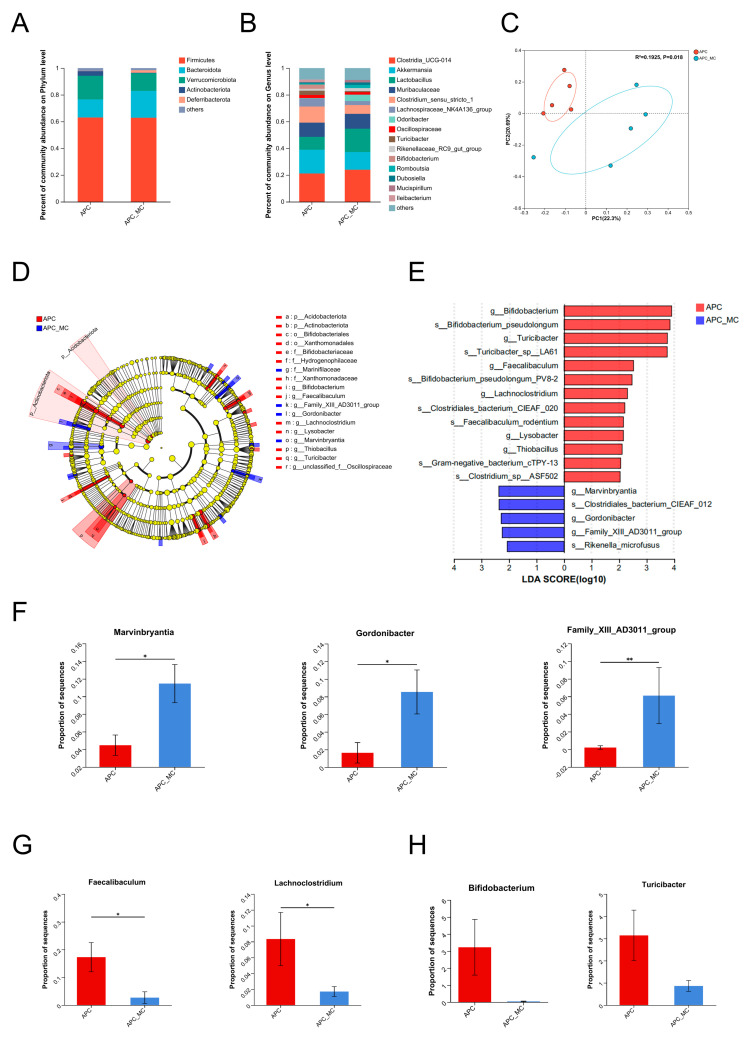
Exposure to MC-LR altered the structure of the gut microbiota. (**A**) Relative abundance of microbial taxa at phylum levels in MC-LR-exposed group and the control group. (**B**) Relative abundance of microbial taxa at genus levels in MC-LR-exposed group and the control group. (**C**) PCoA plots based on the Bray–Curtis distance matrix indicate beta diversity in MC-LR-exposed group and the control group. (**D**) LEfSe cladogram displays the dominant bacteria in the three enterotype subgroups (phylum-to-genus level). Only taxa with an LDA score >  2 are presented. (**E**) The LDA score of the discriminative microbial taxa (genus-to-species level) between MC-LR-exposed and control groups. Only taxa with an LDA score >  2 are presented. (**F**–**H**) Comparisons of the relative abundance of *Marvinbryantia*, *Gordonibacter*, *Family_XIII_AD3011_group*, *Faecalibaculum*, *Lachnoclostridium*, *Bifidobacterium* and *Turicibacter* in MC-LR-exposed and control groups. * *p* < 0.05, ** *p* < 0.01.

At the phylum level, *Firmicutes* (63.0%), *Verrucomicrobiota* (17.7%) and *Bacteroidota* (13.5%) were the three dominant groups in the gut microbial communities (Figure 3A). Over 90% of the sequences were assigned to the 15 most abundant genera (Figure 3B). Through comparing the overall community structure through beta diversity, we could further find out the differential bacteria between the exposure and control groups. The PCoA plot shows that the control group and the exposure group were separated (*p* < 0.05), with PC1 and PC2 explaining 22.3% and 20.69% of the variation, respectively (Figure 3C). For exploring the particular bacterial taxa correlated with MC-LR exposure, we used LEfSe analysis to identify bacteria with differentiation at the phylum and genus level (Figure 3D). Furthermore, LDA scores were used to identify essential differences in bacterial abundance between the two groups (Figure 3E, Table S1). Then, we performed a comparative analysis of the microbial communities. Following MC-LR exposure, the group was distinguished by a higher abundance of *Marvinbryantia*, *Gordonibacter* and *Family_XIII_AD3011_group* at the genus level (Figure 3F). By comparison, the control group demonstrated a dominance of *Faecalibaculum* and *Lachnoclostridium* (Figure 3G, Table S2). It is worth noting that *Bifidobacterium* and *Turicibacter* showed a decreased trend in the MC-LR-exposed group, though without statistical significance (Figure 3H). These findings showed that MC-LR significantly altered the gut microbiota in the APC^min/+^ mice, potentially exacerbating colorectal tumor progression.

### 2.4. MC-LR Exposure Aggravated Microbial-Induced Dysregulation of BA Metabolism

**Figure 4:**
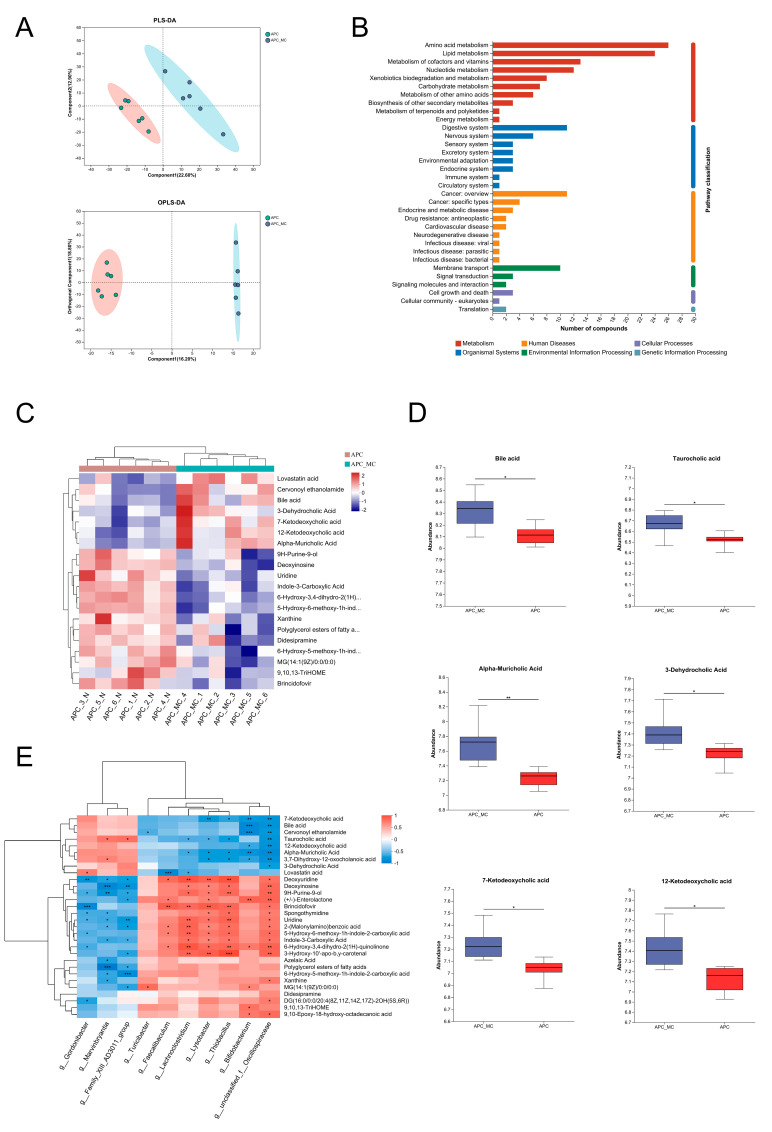
MC-LR exposure aggravated microbial-induced dysregulation of BA metabolism. (**A**) PLS-DA together with OPLS-DA score plots comparing metabolic profile between control and MC-LR-exposed groups. (**B**) KEGG pathway classification: detection and annotation of metabolites. The *x*-axis stands for second-grade items of the KEGG pathway, while the *y*-axis stands for the number of identified metabolites. (**C**) Heat map of the significantly differential metabolites in MC-LR-exposed and control group (*p* < 0.05, VIP >1). (**D**) Relative abundance of differential BAs in MC-LR-exposed and control groups. (**E**) Correlation of differentially altered microbes with metabolites after MC-LR exposure analyzed using Spearman’s correlation. * *p* < 0.05, ** *p* < 0.01, *** *p* < 0.001.

Alterations in the structure of the intestinal flora influence the metabolic phenotype of the host. To assess the effect of MC-LR exposure on the host metabolism, we conducted metabolic profiling of fecal samples obtained from the mice treated with MC-LR and control mice. Partial least squares discrimination analysis (PLS-DA) and orthogonal partial least squares discriminant analysis (OPLS-DA) revealed a remarkable aggregation for similar samples and a significant separation for different groups (Figure 4A). In total, 584 metabolites (*p* < 0.05, VIP > 1) were significantly altered in the intestinal contents of the mice exposed to MC-LR by comparison with the control group (Figure S1). Subsequently, the recognized metabolites were allocated to the KEGG database. These metabolites were categorized into 33 second-grade pathways according to the KEGG classification (Figure 4B). Notably, “amino acid metabolism”, “lipid metabolism”, “digestive system”, “cancer: overview” and “membrane transport” were enriched in each respective first-grade pathway. To visualize the differences in the MC-LR-exposed and control groups, we selected the top 20 differential metabolites in terms of abundance and ran a hierarchical cluster analysis by a heat map. The data show that the BA biosynthesis pathway was significantly affected by the MC-LR exposure (Figure 4C,D). The abundance of five distinct types of BAs were increased significantly after the MC-LR exposure, namely taurocholic acid (TCA), alpha-muricholic acid (α-MCA), 3-dehydrocholic acid (3-DHCA), 7-ketodeoxycholic acid (7-KDCA) and 12-ketodeoxycholic acid (12-KDCA) (Figure 4D, Table S3).

To ascertain the potential association between changes in fecal BA abundance and gut dysbiosis in the APC^min/+^ mice, we conducted Spearman’s correlation analysis between differential bacteria and metabolites. Our result suggested that TCA levels were positively related to the abundance of *Marvinbryantia* and *Family_XIII_AD3011_group*, while they were negatively related to the abundance of *Lachnoclostridium*, *Lysobacter*, *Thiobacillus* and *g_unclassified_f_Oscillospiraceae* (Figure 4E). Moreover, *Bifidobacterium* and *g_unclassified_f_Oscillospiraceae* exhibited a significantly negative relationship with BAs, including α-MCA, 7-KDCA and 12-KDCA. In conclusion, MC-LR exposure led to a significant alteration in fecal BAs, which correlated significantly with MC-LR-induced dysbiosis of the intestinal flora.

## 3. Discussion

MC-LR belongs to the class of toxins formed from cyanobacteria. This toxin is commonly associated with algal blooms and represents a considerable threat to the quality of drinking water [45]. The WHO has emphasized that drinking water concentration of MCs should not be higher than 1 μg/L [46]. This guideline is based on a 13-week oral gavage study of MC-LR in normal mice, which determined the no-observed-adverse-effect level (NOAEL) for the liver to be at a dose of 40 μg/kg body weight/day [47]. A previous study showed that a daily gavage of 80 µg/kg body weight in mice for 28 weeks caused only mild injuries to hepatocytes around the central veins [48]. In another study, male rats exposed to 50 μg/kg body weight of MC-LR in drinking water for 28 days showed no change in the average levels of early markers of hepatotoxicity [49]. These findings support the safe dose of 40 µg/kg body weight/day identified in our study. However, the function of MC-LR in vulnerable groups is not well understood. Therefore, we used conventional transgenic APC^min/+^ mice combined with microbiomics and metabonomics approaches to explore the underlying factors of subchronic exposure to the safety standard dose of MC-LR.

In this research, we illustrated that MC-LR exposure can promote CRC progression in APC^min/+^ mice. We observed that the MC-LR exposure revealed a significant increase in colorectal malignant phenotype and a reduction in colorectal length after 8 weeks of MC-LR exposure. This result is in agreement with a previous finding that oral delivery of MC-LR led to increased colonic mucosal ulcers and shortened colon length in mice with DSS-induced colorectal cancer after a short period (14 days) of exposure at a high dose [20]. In another comparable study, high doses of MC-LR were administered to male C57Bl/6J mice induced with azoxymethane (AOM), revealing significantly larger average areas of abnormal crypt foci in the colon [50]. We also observed that the overall number of goblet cells in the colorectum of the MC-LR-exposed mice was reduced. In the colon, epithelial goblet cells secrete mucus that acts as an antimicrobial physical barrier against bacteria [51]. The loss of goblet cells disrupts the mucus layer, resulting in greater bacterial translocation into the intestinal mucosa [52]. The results showed that exposure to MC-LR promoted colorectal tumor progression and impaired intestinal barrier function in the APC^min/+^ mice, which has not been previously reported in other studies. This important finding further emphasizes the necessity to be cautious about the safety doses of MC-LR, as it may still cause damage to the colorectum.

Gut microbiota dysbiosis is a major contributor to the incidence and development of colorectal cancer [53,54]. Many studies have shown that prolonged contact with MC-LR leads to notable alterations in the gut microbiota composition. One study found that mice exposed to MC-LR exhibited a significant increase in *Turicibacter* and *Clostridium* levels while showing a substantial decrease in *Ruminococcus* and *Alloprevotella* levels [36]. Another study showed that a decrease in *Actinobacteria* and *Saccharibacteria* levels was observed in mice exposed to MC-LR [55]. However, most studies have used normal rather than cancer-prone mice. In this study, we provided evidence that MC-LR exposure may promote CRC progression by altering the composition of gut microbiota and causing gut microbiota dysbiosis in APC^min/+^ mice. Specifically, we found that in APC^min/+^ mice with subchronic exposure to MC-LR, the abundance of *Marvinbryantia*, *Gordonibacter* and *Family_XIII_AD3011_group* was increased, while that of *Faecalibaculum*, *Lachnoclostridium*, *Bifidobacterium* and *Turicibacter* was significantly decreased. *Marvinbryantia* was reported to be positively correlated with the risk of gastroduodenal ulcers [56]. *Gordonibacter* was increased in Western-style-diet-induced mice, which contributed to metabolic-associated fatty liver disease [57]. *Family_XIII_AD3011_group*, which was increased in a benzene-exposed group, was highly related to hematopoietic toxicity and IL-5 [58]. On the other hand, the abundance of four probiotics, i.e., *Faecalibaculum*, *Lachnoclostridium*, *Bifidobacterium* and *Turicibacter*, was depleted in the MC-LR-exposed group. It is widely known that these gut probiotics are reduced when an imbalance occurs in the gut flora [53,54,59]. Taken together, our results indicated that the development of CRC induced by MC-LR was at least partially attributed to a gain of pathobionts and a loss of protective bacteria.

**Figure 5:**
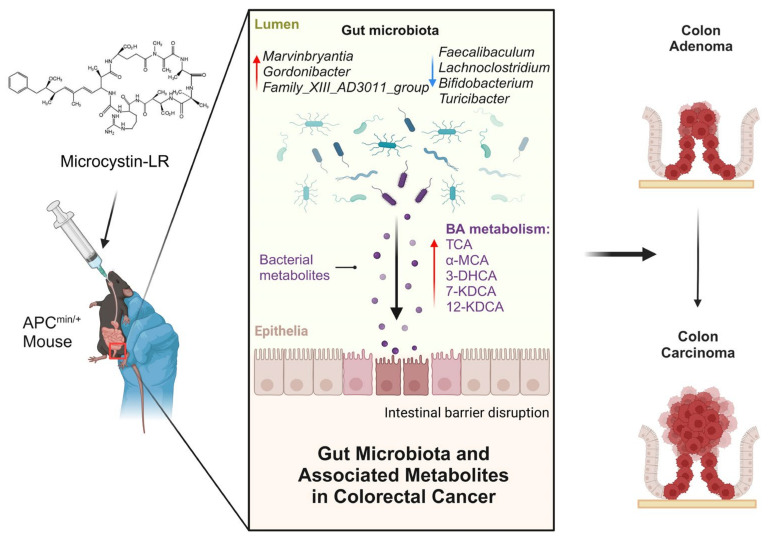
Mechanisms by which MC-LR promotes CRC progression. Figure was created with elements from BioRender.com, accessed on 25 April 2024.

It was also observed that the lipid metabolism was the second most significantly enriched metabolic pathway in the mice exposed to MC-LR, which included BA biosynthesis. Primary bile acids are essential for lipid digestion, cholesterol metabolism, host–microbe interactions and regulatory pathways within the host. Although the majority of BAs are reabsorbed and recycled through the enterohepatic circulation, approximately 5% are transformed in the intestinal tract [60]. Non-reabsorbed bile acids may act as metabolic substrates for microbes, transforming into secondary bile acids [61]. The interactions among the host, BAs and the gut microbiota can impact various cancers, including colorectal cancer [60,62,63]. We confirmed a significant increase in the amounts of BAs in the intestinal contents of the APC^min/+^ mice exposed to MC-LR. Previous studies have shown that reduced concentrations of taurocholate and taurodeoxycholic acid, coupled with increased taurine levels, suggest that MC-LR could influence bile acid metabolic processes [64]. Subsequently, we also noted that the metabolite changes were significantly related to the changes in gut microbial abundance, with TCA having the most positive correlation with *Marvinbryantia* and *Family_XIII_AD3011_group*. The result is supported by previous studies [65,66]. At present, there is a lack of research reports on these two bacteria, and how they regulate bile acid metabolism is still unclear, which needs further study. However, TCA was found to be positively correlated with the risk of colon cancer [67]. Furthermore, increased levels of secondary bile acids in the colon can trigger cellular responses, such as the activation of the Wnt/β-catenin and NF-κB signaling pathways, resulting in DNA oxidative harm and enhanced mitotic activities [68,69]. Overall, MC-LR may induce specific bacteria to participate in the synthesis of BAs, affecting the concentration of TCA or other BAs in the intestine, thereby promoting the severity of colorectal tumor (Figure 5).

## 4. Conclusions

In summary, this is the first attempt to study the safe dose of MC-LR in cancer-susceptible mice from a multi-omics approach. The present study indicated that exposure to MC-LR promotes CRC progression by modulating the composition of the gut microbiota in APC^min/+^ mice. The gut microbiota dysbiosis induced by MC-LR could increase BA levels in the colorectum, potentially serving as the key mechanism in promoting cancer. Therefore, more stringent restrictions must be adopted for MC-LR in finished drinking water to protect vulnerable populations from its toxicity, especially those with pre-existing intestinal diseases.

## 5. Materials and Methods

### 5.1. Animals and Experimental Design

SPF male APC^min/+^ C57BL/6 mice (aged 6–7 weeks, 20–22 g) were obtained from GemPharmatech Co., Ltd. (Nanjing, China) and maintained at the Animal Core Facility of Nanjing Medical University. The mice were nourished with a standard rodent diet and maintained in a controlled environment with a 12:12 h light–dark cycle. After adapting to the environment for 2 weeks, 24 mice were randomly and equally assigned to two groups: a treatment group (gavage with 40 µg/kg MC-LR dissolved in 0.9% NaCl solution) and a control group (gavage with the same 0.9% NaCl solution based on mice body weight). MC-LR, with a purity exceeding 95%, was procured from Enzo Life Sciences (Farmingdale, NY, USA). The mice received either the 0.9% NaCl solution or MC-LR by oral gavage for 8 weeks. Body weights were measured daily.

### 5.2. Sample and Data Collection

Prior to sacrificing the mice, fresh fecal samples were collected in sterile tubes and promptly placed on dry ice. At the time of sacrifice, the intestines were longitudinally opened along the mesenteric margin and rinsed with cold PBS. Intestinal length was measured, and the intestinal contents were collected in sterile tubes. Tumors were counted and measured for tumor load, calculated as the sum of the mean diameters of all tumors in each mouse (mean diameter = [major diameter + minor diameter]/2) [70]. The fecal samples were stored at −80 °C until needed, and the colon tissues were fixed in 4% paraformaldehyde (PFA).

### 5.3. Histopathological Examination

Colon tissues were embedded in paraffin, sectioned and subjected to staining with hematoxylin and eosin (H&E) as well as periodic acid–Schiff (PAS). A PAS dye solution set (Servicebio, Wuhan, China) was employed for the PAS staining, following the instructions provided by the manufacturer. Microscope inspection, image acquisition and analysis of the sections were conducted by a pathologist blinded to the groups. The Image-Pro Plus software (version 6.0, Media Cybernetics Corporation, Rockville, MD, USA) was utilized to quantify the tumor area.

### 5.4. Full-Length 16S Sequencing and Data Processing

Six fecal samples were randomly selected from each of the control and treatment groups, and genomic DNA from bacterial species was extracted using a PF Mag-Bind Stool DNA Kit (Omega Bio-tek, Norcross, GA, USA) in accordance with the manufacturer’s stipulations. Subsequently, the concentration and purity of the DNA were assessed using a NanoDrop 2000 UV–vis spectrophotometer (Thermo Scientific, Wilmington, NC, USA). Amplification of full-length (V1–V9) bacterial 16S rRNA gene sequences was achieved using primers 27F (AGRGTTYGATYMTGGCTCAG) and 1492R (RGYTACCTTGTTACGACTT) through a thermocycler PCR system (GeneAmp 9700, ABI, Hampton, NH, USA). After amplification, quantification of the amplicons was performed, and the resulting equimolar concentrations were normalized before amalgamation. Subsequently, the combined samples underwent sequencing on a PacBio Sequel IIe System (Pacific Biosciences, Menlo Park, CA, USA). Detailed data analysis is available in the Supplementary Information S1.1.

### 5.5. Biodiversity Analysis

Samples with poor correlation between the same group were excluded, and finally, 5 groups of samples from each group participated in the subsequent analysis. Alpha diversity assessment was performed to elucidate species diversity within each sample, with the calculations executed using Mothur v1.30.2. Beta diversity calculations utilized principal coordinate analysis (PCoA) based on the Bray–Curtis dissimilarity, executed with the Vegan v2.4.3 package. Comparisons of bacterial abundance and diversity were conducted using the Mann–Whitney U test. The linear discriminant analysis (LDA) effect size (LEfSe) [71] (http://huttenhower.sph.harvard.edu/LEfSe, accessed on 28 November 2022) was performed to identify the significantly abundant taxa (phylum to species) of bacteria between the different groups (LDA score > 2, *p* < 0.05). The bioinformatics analysis in this study was conducted with the assistance of the Majorbio Cloud Platform (www.majorbio.com).

### 5.6. Metabolomic Profiling and Data Analysis

The 6 intestinal contents corresponding to 16S sequencing were carefully placed into a 2 mL centrifuge tube, accompanied by the addition of a 6 mm diameter grinding bead. For metabolite extraction, 400 μL of a solution composed of methanol and water in a 4:1 (*v*:*v*) ratio, containing 0.02 mg/mL of the internal standard (L-2-chlorophenylalanine), was employed. Subsequently, the resulting supernatant was carefully transferred to the injection vial for subsequent LC-MS/MS analysis.

The quality control (QC) samples were methodically dispensed and scrutinized using identical procedures as the analytic samples. Periodic injections were implemented at established intervals to systematically assess the stability of the analysis. The analysis was performed using a SCIEX UPLC-Triple TOF 5600 system equipped with an ACQUITY HSS T3 column (100 mm × 2.1 mm i.d., 1.8 μm; Waters, Milford, MA, USA) at Majorbio Bio-Pharm Technology Co., Ltd. (Shanghai, China). The UPLC system was coupled to a quadrupole-time-of-flight mass spectrometer (TripleTOFTM5600+, Sciex, Framingham, MA, USA) equipped with an electrospray ionization (ESI) source operating in both positive and negative modes. Data acquisition was executed in the information-dependent acquisition (IDA) mode, spanning a mass range of 50–1000 *m*/*z*. A detailed description of the data analysis is provided in the Supplementary Materials.

### 5.7. Statistical Analysis

Data are presented as the mean ± standard error of the mean (SEM) and were subjected to comparison through unpaired Student’s *t*-test or the Mann–Whitney U test between two groups, as appropriate. Statistical significance was attributed to comparisons with *p* < 0.05. All statistical analyses were performed using GraphPad Prism, version 9.0 (GraphPad, La Jolla, CA, USA), or the R package.

## Data Availability

Data will be made available on request.

## Supplementary Materials

The following supporting information can be downloaded at: https://www.mdpi.com/article/10.3390/toxins16050212/s1, Figure S1: Volcano plot of differential metabolites in the intestinal contents of mice treated with MC-LR compared to the control group; Table S1: Table of linear discriminant analysis results; Table S2: The differential bacteria between MC-LR exposure and control; Table S3: The differential BAs in MC-LR-exposed mice compared with control mice.
