# Supplementary material for: Exposure to Microcystin-LR Promotes Colorectal Cancer Progression by Altering Gut Microbiota and Associated Metabolites in APCmin/+ Mice
Source: Toxins (Basel). 2024 Apr 30;16(5):212. doi: 10.3390/toxins16050212 (PMC11125743; doi:10.3390/toxins16050212)
Supplement: Supplementary file 1 [file toxins-16-00212-s001.zip › toxins-2949173-supplementary.pdf]

Exposure to Microcystin-LR Promotes Colorectal Cancer Progression by Altering Gut Microbiota and Associated Metabolites in $APC^{min/+}$ Mice

Yuechi Song, Xiaochang Wang, Xiaohui Lu and Ting Wang

Supplemental materials and methods

1.1. Detailed data analysis about gut microbiota

PacBio raw reads underwent processing utilizing the SMRTLink analysis software (version 11.0) to acquire high-quality Hifi reads, ensuring a minimum of three complete passes and a 99% sequence accuracy. Subsequently, the Hifi reads underwent barcode identification and length filtration. In the context of the bacterial 16S rRNA gene, sequences with a length <1,000 or >1,800 bp were systematically eliminated. The Hifi reads were then subjected to clustering into operational taxonomic units (OTUs) through UPARSE 11, employing a 97% sequence similarity threshold. To minimize the effects of sequencing depth on alpha and beta diversity measure, the number of 16S rRNA gene sequences from each sample were rarefied to 8205. The taxonomic classification of each OTU representative sequence was carried out using RDP Classifier version 2.13 against the 16S rRNA gene database (Silva v138), employing a confidence threshold of 0.7.

1.2. *Detailed data analysis about metabolomic profiling*

The pretreatment of LC/MS raw data was performed by Progenesis QI (Waters Corporation, Milford, USA) software, and a three-dimensional data matrix in CSV

format was exported. The information in this three-dimensional matrix included: sample information, metabolite name and mass spectral response intensity. Internal standard peaks, as well as any known false positive peaks (including noise, column bleed, and derivatized reagent peaks), were removed from the data matrix, de-redundant and peak pooled. At the same time, the metabolites were identified by searching database, and the main databases were the HMDB (http://www.hmdb.ca/), Metlin (https://metlin.scripps.edu/) and Majorbio Database.

The data underwent analysis using the freely accessible online platform of Majorbio Cloud (cloud.majorbio.com). Metabolic features detected in no less than 80% of any set of samples were preserved. Following filtration, minimal metabolite values were imputed for specific samples in which the metabolite levels descended below the lower limit of quantitation. Subsequently, each metabolic feature underwent normalization by summation. To mitigate errors stemming from sample preparation and instrument instability, the response intensity of the sample mass spectrum peaks was normalized using the sum normalization method, culminating in the acquisition of the normalized data matrix. Simultaneously, variables exhibiting a relative standard deviation (RSD) greater than 30% of QC samples were excluded, and a logarithmic base 10 transformation was performed to derive the definitive data matrix for subsequent analysis.

The R package “ropls” (Version 1.6.2) was utilized for principal component analysis (PCA) and orthogonal least partial squares discriminant analysis (OPLS-DA), with a 7-cycle interactive validation for assessing model stability. Metabolites with VIP >

1, $P < 0.05$ were identified as significantly different metabolites based on the Variable Importance in the Projection (VIP) obtained from the OPLS-DA model and the p-value generated by a student's t-test. Data analysis encompassed multivariate statistical analysis, selection, and correlation of differential metabolites, GO and KEGG pathway enrichment. The association between differentially altered bacteria and metabolites was computed using Spearman rank correlation coefficients, and heatmaps were generated in the R platform (version 4.1.3).

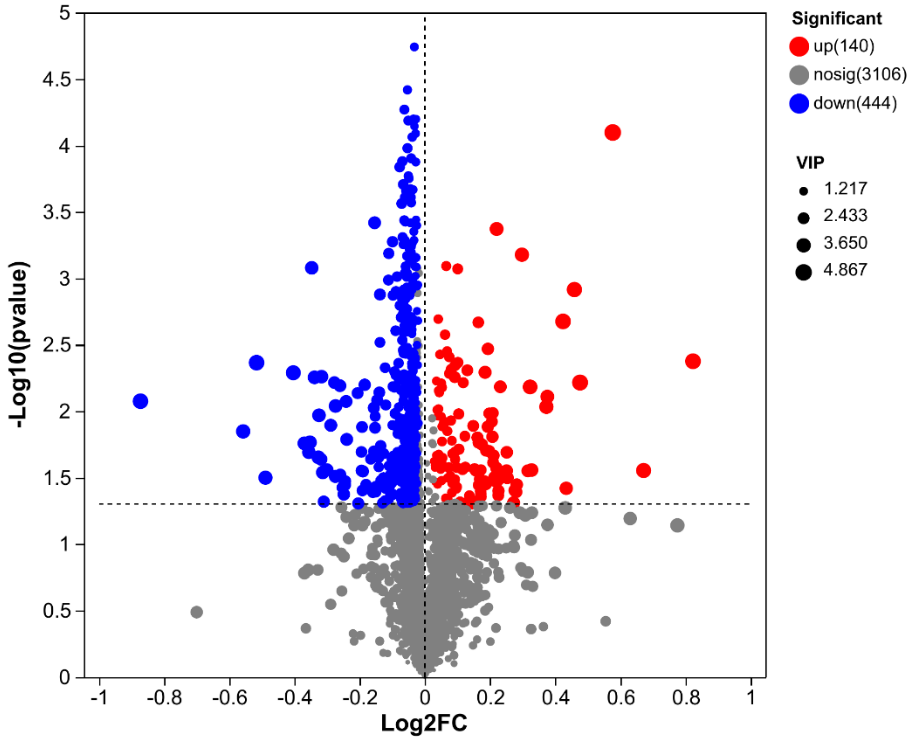

Figure S1. Volcano plot of differential metabolites in the intestinal contents of mice treated with MC-LR compared to the control group.

Table S1. Table of linear discriminant analysis results.

Species name	Group	LDA value	*P* value
g__Bifidobacterium	APC	3.905	0.043
g__Bifidobacterium.s__Bifidobacterium_pseudolongum	APC	3.846	0.043
g__Turicibacter	APC	3.750	0.045
g__Turicibacter.s__Turicibacter_sp__LA61	APC	3.745	0.045
g__Bifidobacterium.s__unclassified	APC	2.862	0.032
g__unclassified_f__Oscillospiraceae.s__unclassified	APC	2.525	0.006
g__unclassified_f__Oscillospiraceae	APC	2.525	0.006
g__Faecalibaculum	APC	2.523	0.017
g__Lachnoclostridium.s__unclassified	APC	2.469	0.004
g__Bifidobacterium.s__Bifidobacterium_pseudolongum_PV8-2	APC	2.456	0.013
g__Lachnoclostridium	APC	2.301	0.045
o__Clostridia_UCG-014.s__uncultured_Escherichia_sp	APC	2.256	0.011
g__Faecalibaculum.s__unclassified	APC	2.251	0.027
g__Lachnospiraceae_NK4A136_group.s__Clostridiales_bacterium_CIEAF_020	APC	2.205	0.022
g__Lachnoclostridium.s__uncultured_bacterium	APC	2.160	0.050
g__Faecalibaculum.s__Faecalibaculum_rodentium	APC	2.156	0.035
g__Lysobacter	APC	2.154	0.013
g__Thiobacillus	APC	2.109	0.013
g__Thiobacillus.s__uncultured_bacterium	APC	2.057	0.013
g__norank_f__Muribaculaceae.s__Gram-negative_bacterium_cTPY-13	APC	2.056	0.028
g__Lachnospiraceae_UCG-006.s__Clostridium_sp__ASF502	APC	2.035	0.034
o__Clostridia_UCG-014.s__uncultured_rumen_bacterium	APC_MC	3.277	0.011
g__Odoribacter.s__unclassified	APC_MC	2.805	0.044
g__Marvinbryantia	APC_MC	2.368	0.028
g__Marvinbryantia.s__Clostridiales_bacterium_CIEAF_012	APC_MC	2.361	0.028
g__Gordonibacter	APC_MC	2.285	0.027
g__Gordonibacter.s__uncultured_bacterium	APC_MC	2.270	0.027
g__Family_XIII_AD3011_group	APC_MC	2.246	0.007
f__Eubacterium_coprostanoligenes_group.s__unclassified	APC_MC	2.235	0.037
g__Family_XIII_AD3011_group.s__uncultured_bacterium	APC_MC	2.209	0.003
g__Staphylococcus.s__unclassified	APC_MC	2.199	0.037
g__Rikenella.s__Rikenella_microfusus	APC_MC	2.065	0.049

Table S2. The differential bacteria between MC-LR exposure and control groups.

Species name	APC_Mean(%)	APC_MC_Mean(%)	*P* value
g__unclassified_f__Oscillospiraceae	0.2011	0.07556	0.007686
g__Family_XIII_AD3011_group	0.002031	0.06094	0.00902
g__Thiobacillus	0.01422	0	0.01543
g__Lysobacter	0.01828	0	0.01572
g__Faecalibaculum	0.1727	0.02681	0.02127
g__Gordonibacter	0.01625	0.08531	0.03286
g__Marvinbryantia	0.04469	0.1146	0.03327
g__Lachnoclostridium	0.08328	0.01706	0.04172

Table S3. The differential BAs in MC-LR exposed mice compared with control mice.

Metab ID	Metabolite	Regulate	VIP	*P* value
metab_10126	Alpha-Muricholic Acid	up	2.3489	0.005552
metab_2888	7-Ketodeoxycholic acid	up	1.5108	0.01084
metab_2767	12-Ketodeoxycholic acid	up	1.8354	0.01211
metab_12167	Bile acid	up	1.2479	0.02151
metab_1389	Taurocholic acid	up	1.223	0.02549
metab_16468	7-Sulfocholic acid	up	2.6446	0.02677
metab_14975	Glycochenodeoxycholic acid 3-glucuronide	up	3.0679	0.03809
